# A pilot heat-health warning system co-designed for a subtropical city

**DOI:** 10.1371/journal.pone.0294281

**Published:** 2023-11-10

**Authors:** Shih-Chun Candice Lung, Ming-Lone Liou, Jou-Chen Joy Yeh, Jing-Shiang Hwang

**Affiliations:** 1 Research Center for Environmental Changes, Academia Sinica, Taipei, Taiwan; 2 Department of Atmospheric Sciences, National Taiwan University, Taipei, Taiwan; 3 Graduate Institute of Environmental Engineering, National Taiwan University, Taipei, Taiwan; 4 Institute of Statistical Science, Academia Sinica, Taipei, Taiwan; Al Mansour University College-Baghdad-Iraq, IRAQ

## Abstract

Significant heat-related casualties underlie the urgency of establishing a heat-health warning system (HHWS). This paper presents an evidence-based pilot HHWS developed for Taipei City, Taiwan, through a co-design process engaging stakeholders. In the co-design process, policy concerns related to biometeorology, epidemiology and public health, and risk communication aspects were identified, with knowledge gaps being filled by subsequent findings. The biometeorological results revealed that Taipei residents were exposed to wet-bulb globe temperature (WBGT) levels of health concern for at least 100 days in 2016. The hot spots and periods identified using WBGT would be missed out if using temperature, underlining the importance of adopting an appropriate heat indicator. Significant increases in heat-related emergency were found in Taipei at WBGT exceeding 36°C with reference-adjusted risk ratio (RaRR) of 2.42, taking 30°C as the reference; and residents aged 0–14 had the highest risk enhancement (RaRR = 7.70). As for risk communication, occurring frequency was evaluated to avoid too frequent warnings, which would numb the public and exhaust resources. After integrating knowledge and reconciling the different preferences and perspectives, the pilot HHWS was co-implemented in 2018 by the science team and Taipei City officials; accompanying responsive measures were formulated for execution by ten city government departments/offices. The results of this pilot served as a useful reference for establishing a nationwide heat-alert app in 2021/2022. The lessons learnt during the interactive co-design processes provide valuable insights for establishing HHWSs worldwide.

## Introduction

In 2022, extreme weather events under the trend of climate change have caused thousands of deaths in the US, Europe, and Asia [1]. In order to reduce the significant impacts brought by global environmental changes [2] and to meet sustainability challenges, new ways of transforming knowledge to assist decision making are required [3]. Under the support of major international organizations such as the International Science Council and the United Nations Environment Programme, a global research platform, Future Earth, was officially inaugurated in June 2012 at Rio +20 [4, 5]. Designed to provide the knowledge needed to facilitate transformations toward sustainability, Future Earth promotes transdisciplinary collaboration among natural and social scientists, practitioners, and societal stakeholders and facilitates initiation of new approaches to co-designing solution-oriented research for sustainable development [4]. In addition to being a core principle of Future Earth [6], co-production of knowledge with stakeholders in transdisciplinary research is the key to making a real impact on society via research findings [7], and framing research questions with key stakeholders aiming for solutions is the first step [4]. Thus, under a climate emergency [8], co-designing research with stakeholder engagement is viewed as a promising way for transforming knowledge to assist decision making in tackling sustainability challenges [9].

With the aim of providing solutions, it is crucial to not only integrate evidence and knowledge during iterative discussions among the involved multidisciplinary scientists and stakeholders but also reconcile different preferences and perspectives in the co-design process. Nevertheless, apart from a book released in 2021 [7], very few publications have presented successful cases of “co-designed” research or explored the essentials for carrying out a successful co-design project. This paper showcases a co-designed pilot heat-health warning system (HHWS) for a subtropical city to fill this knowledge gap.

The significant worldwide heat-related casualties in recent years have highlighted the urgency and importance of establishing an effective HHWS [1, 2, 10–17]. Even with the Heatwave Plan for England, Thompson et al. still found there was excess mortality during the heatwave of 2020 in the UK [12]. Franklin et al. assessed health impacts during the heatwaves of 2010–2019 in Queensland and found that all-cause mortality varied considerably due to local demographic and environmental conditions [13]. In addition to local conditions, the choices of an appropriate heat indicator and alert thresholds for the HHWS are critical to ensuring effective protection of the general public [18]. For most cases of HHWSs, either temperature alone or a composite indicator considering both temperature and humidity have been selected for use as a heat indicator [19]. For example, maximum temperature was used in the warning system of the UK, while the US and Canada used the heat index and humidex, respectively, with temperature and humidity taken into consideration [20, 21]. When reviewing the HHWSs of 16 European countries, Casanueva et al. [19] found diverse indicators were adopted along with thresholds that had varied definitions. Different from the majority of indicators considering temperature alone, the systems in Germany and Austria took into account temperature, radiation, wind, and humidity. While most thresholds were defined according to epidemiological evidence, some referred to climatological percentiles [19]. Moreover, for the HHWS developed in Japan, Hong Kong, and Australia, wet-bulb globe temperature (WBGT) was used as one of the indicators [14, 22–24]. For example, the Japanese government launched “Heatstroke Alert” in 2020, wherein an alert is issued when the daily maximum WBGT is expected to reach or exceed 33°C [14]. Establishing an HHWS based on appropriate indicators involves multiple policy considerations which are better addressed by transdisciplinary collaboration of policy makers and scientists in the co-design process. As a demonstration, a co-designed pilot HHWS is presented in this paper with an appropriate heat indicator selected on the basis of scientific evidence with the consensus of policy makers and a multidisciplinary science team.

The fact that any developed HHWS should be region-specific was acknowledged by the Guidance on Warning-System Development put forward by the World Meteorological Organization (WMO) and the World Health Organization (WHO) [25]. In addition, different policy concerns were highlighted in this guidance, including biometeorology, epidemiology and public health, and risk communication, which were given due consideration in our co-design process as detailed in the Materials and Methods section. In general, the weather agency of the central government is responsible for developing a nationwide HHWS. To effectively reduce health risks, responsive plans have to be formulated and carried out by the concerned authorities. All these involve substantial resources. Hence, a city-level pilot HHWS that takes into account all policy concerns but requires less resources would be desirable for evaluating its feasibility as well as identifying knowledge and implementation gaps for the eventual nationwide system. Moreover, communication channels at the city-level are better streamlined, as different departments within a city government are all under the leadership of the mayor, while those at the country-level are more complicated as the authorities in the central government responsible for various responsive plans may have multiple superiors. Lessons learnt from city-level development and implementation in the pilot work are expected to provide useful references for establishing an effective nationwide HHWS.

In the city-level pilot work presented here, WBGT was chosen as the heat indicator for multiple reasons. As a weighted combination of dry-bulb temperature, natural wet-bulb temperature, and globe temperature, WBGT considers four heat-stress relevant meteorological parameters, including temperature, relative humidity, wind speed, and solar radiation [26]. WBGT is also consistent with our daily experience of feeling hot when exposed to solar radiation and feeling cooler under high wind. Moreover, the association of WBGT with heat-health impacts of workers has been reported in numerous studies [26–31]. For the past 60 years, occupational agencies worldwide have adopted WBGT as the heat stress indicator for protecting workers’ health [32]. Recent studies have also shown the association of increased WBGT with heat-health impacts of the general public [33–38]. For example, Nakamura et al. found a robust association between WBGT and heatstroke incidence at the daily maximum WBGT of 33°C in all 47 prefectures of Japan in 2015–2019 [37]. Ueno et al. also found a significant relationship between WBGT and heat-related ambulance transport in 2017–2020 in Japan [38]. In short, WBGT is a preferred choice for an HHWS because it is a physiological-based indicator representing human heat stress, while most other indicators are based on the subjective perceptions of human comfort, such as apparent temperature [39]. The purpose of an HHWS is to reduce heat-health risks rather than enhance perceived comfort [19, 39, 40].

The objective of this paper is to showcase an evidence-based pilot HHWS developed for Taipei City, Taiwan, through a co-design process, which is presented to highlight the viewpoints and preferences of policy makers. The scientific findings are also summarized to address the policy concerns corresponding to the aspects of biometeorology, epidemiology and public health, and risk communication identified in the co-design process. The overall considerations in integrating scientific evidence with policy concerns from different perspectives are also presented. With certain detours, the knowledge acquired from this pilot work of 2018 has laid a foundation for the nationwide heat-alert app developed in 2021/2022, demonstrating the difficulty involved in transforming knowledge into actions for tackling the real-world challenge of sustainability. Lessons learnt in the process can be applied to other countries and facilitate the implementation of HHWSs for reducing health risks to meet the United Nation Sustainable Development Goal 3 (SDG3), ensure healthy lives and promote well-being for all at all ages [41]. The essential elements for carrying out a successful co-design project, as examined in this research, can also help expedite the execution of co-designed projects to tackle other SDG challenges. To our best knowledge, our publication is the first to describe an interactive co-design process for establishing a city-level pilot HHWS. The present case serves as an example in transdisciplinary research for translating knowledge into action to tackle SDG challenges.

## Materials and methods

The co-design process involving stakeholder engagement, the methodology for addressing three aspects of the policy concerns of this pilot HHWS, and the implementation of this pilot HHWS along with the planning of responsive actions are described in this section.

### Stakeholder engagement

Two key stakeholders were involved in this process, one of which is the Central Weather Bureau (CWB), with the mandate of developing the nationwide HHWS. In view of the higher mortality and morbidity on hot days in Taiwan [33, 35, 42], the CWB has been deliberating on the establishment of a national HHWS. Scientific evidence has been presented to the CWB officials by our team since 2012, supporting WBGT as the preferred heat indicator. Typically, an HHWS warning is issued based on forecasts for the following days. WBGT forecast involves estimating solar radiation (projecting cloud formation and movement) which is challenging; thus establishing a HHWS with WBGT as the heat indicator was shelved by the CWB.

The other stakeholder is the Department of Environmental Protection of Taipei City Government (TDEP), which is responsible for reducing impacts of climate change. In 2017, upon learning about WBGT from the CWB, the TDEP officials initiated discussion with our team and were later convinced of the suitability of WBGT as the heat indicator for an HHWS, especially for people engaged in outdoor activities. A pilot HHWS was thus developed for Taipei City (abbreviated as “TPE-pHHWS”), with science-based criteria and a mechanism set up for triggering responsive programs within the Taipei City Government to reduce heat-health risks. Furthermore, the underlying anticipated benefit is to gain knowledge that may induce and facilitate the CWB to establish a national WBGT-based HHWS.

**Fig 1:**
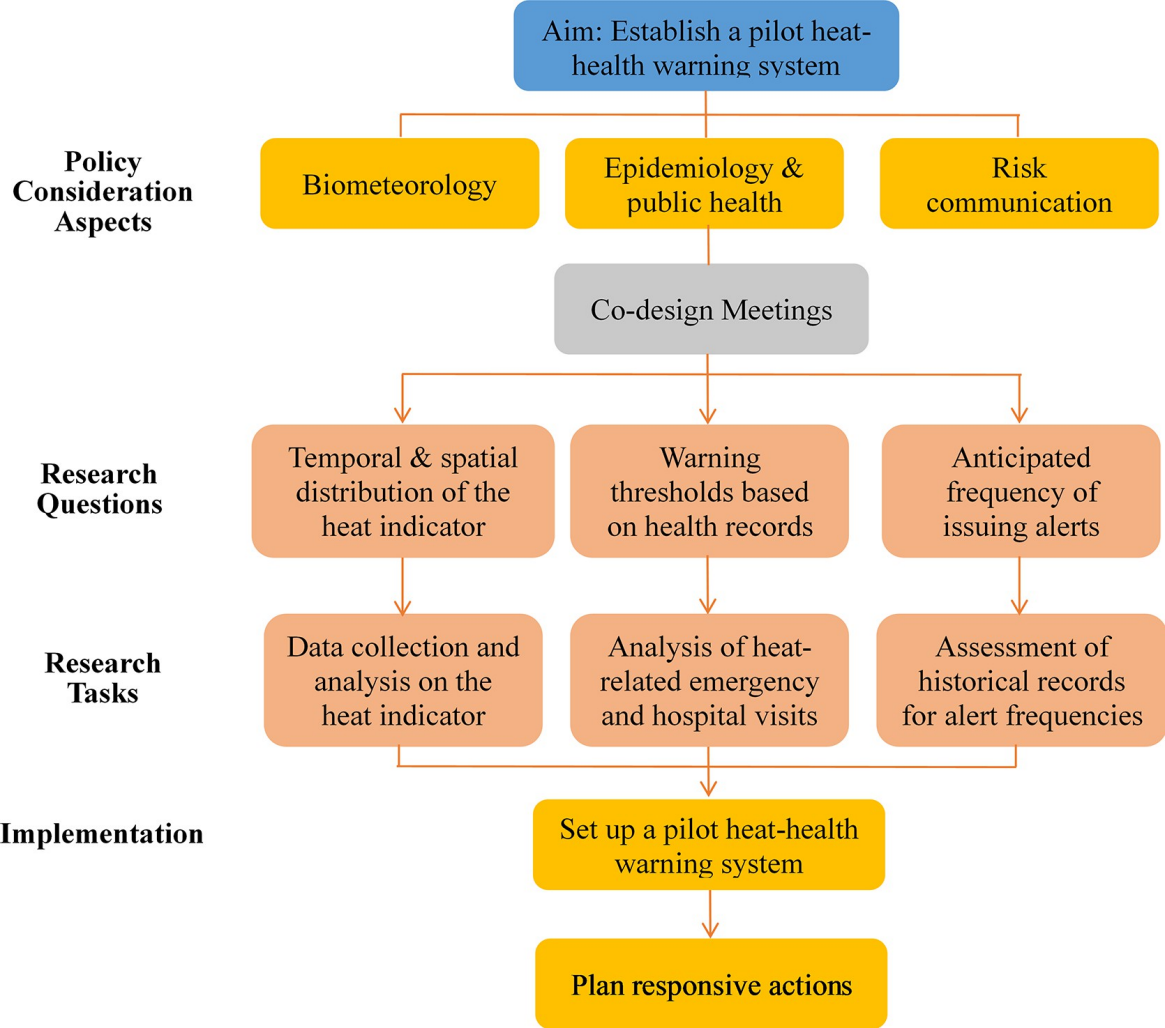
Framework of co-designing a pilot heat-health warning system involving a multidisciplinary science team and policy makers.

The knowledge gaps were identified as the first step in developing this WBGT-based TPE-pHHWS. The major policy concerns regarding three aspects of warning-system development emphasized by WMO and WHO, namely, biometeorological, epidemiological and public health, and risk communication, are shown in Fig 1 [25]. After several rounds of discussion, the following three corresponding research questions were established: (1) whether heat stress levels as measured by the adopted heat indicator, WBGT, reached/reach health-concerning levels; (2) what is the appropriate threshold for issuing heat-health warnings according to local evidence; and (3) what is the optimal frequency for warnings to be issued such that responsible government agencies, private sectors, and the general public take heed of these warnings. As shown in Fig 1, addressing these concerns requires (1) biometeorological evidence on the temporal and spatial distribution of WBGT in Taipei City, (2) epidemiological findings from local health records for selecting a WBGT threshold for triggering responsive programs, and (3) the frequency of alert issuance estimated from historical records. The research tasks detailed in the following subsections were carried out by our multidisciplinary team composed of experts in environmental science, statistics, and public health. The TPE-pHHWS thus developed was co-implemented by our science team and the TDEP, while responsive actions were planned and carried out mainly by the city officials as described at the end of this section.

### Biometeorological concern

Historical data were assessed, and monitoring was carried out to evaluate WBGT in Taipei City. WBGT at workplaces was traditionally measured using a monitor such as QuesTemp (3M^TM^, Oconomowoc, WI, USA), which is not waterproof and thus not suitable for long-term outdoor monitoring. Instead, our team obtained WBGT using equations derived by scientists in the US national laboratories according to fundamental principles of heat and mass transfer with inputs of routine meteorological measurements, which has been validated with monitoring [43]. The temporal WBGT trend during 2002–2016 was evaluated using hourly data from the only standard CWB station in Taipei City. The highest WBGT category in Japan (≥ 31°C; Japanese Ministry of the Environment [23]) classified as “dangerous” to the general public and that in the US as “hazardous (black flag)” to workers’ health (> 32.2°C; Air Force USA [44]) were adopted to assess whether WBGT reached health-concerning levels.

To obtain the spatial distribution of WBGT, four HOBO stations (HOBO, RX3003, Onset, Bourne, MA, USA) were set up because there was only one standard CWB station in Taipei City. Contrary to standard CWB stations, which are situated on grassland, the HOBO stations were set up on concrete surfaces, the type of surface most commonly encountered by residents living in Taipei, to assess the actual heat stress exposure of residents. Meteorological observations of one-min resolution were wirelessly transmitted to the database; the calculated WBGT values were then shown on the internal TDEP webpage with a lead time of 10–15 minutes. WBGT during warm seasons, defined as from May to October according to Taiwan’s climate, of 2017 and 2018 were planned to be collected.

Moreover, it was found in 2018 that the Department of Education of Taipei City Government (TDOE) collaborated with the CWB in setting up basic weather stations (Vantage Pro, Davis Instruments, Hayward, CA, USA) for monitoring temperature, relative humidity, wind direction, and wind speed in most elementary schools in Taipei for education purposes (Taipei Weather Inquiry-based learning Network, TWIN) (http://weather.yaes.tp.edu.tw/index.htm). Most of these TWIN stations are situated on rooftops and not grassland. TWIN data were obtained through the TDOE with a lead time of 2–3 months. After discarding 4 schools with a high percentage of missing data, meteorological measurements of 57 schools were analyzed (S1 Fig). TWIN did not have solar radiation measurements; hence, solar radiation from the CWB station located in Taipei City was adopted to calculate WBGT.

The difference in HHWS using WBGT compared to that with temperature alone was assessed by showing the difference in the spatial and temporal distributions of temperature and WBGT. Inverse distance weighting extrapolation was used for these two indicators to illustrate their spatial variations in Taipei City. The hot periods in the diurnal cycle during the monitoring periods were also identified for temperature and WBGT to demonstrate differences in their temporal distribution.

### Epidemiological and public health concern

For alerts to be issued, an appropriate WBGT threshold has to be determined. The consensus reached in the co-design process was to use epidemiological evidence of heat-related emergency and hospital visits for prevention purposes because morbidity always precedes mortality. The methodology for identifying an appropriate threshold for an HHWS with the data for the whole of Taiwan has been reported [33, 36]. Taipei City is located in a basin where heat accumulates more easily than in most of the regions in Taiwan; thus, WBGT–health relationships were assessed using the same methodology for the Taipei metropolitan area, comprising both Taipei City and New Taipei City. On the one hand, both cities are located in the Taipei basin, experiencing similar weather conditions. On the other hand, residents in New Taipei City would visit hospitals in Taipei City and vice versa, thus making it difficult to differentiate between cases from the two cities.

The relationships of WBGT with heat-related emergency or hospital visits were examined using generalized additive models with Poisson distribution. The daily maximum WBGT of the two CWB stations in the Taipei metropolitan area were averaged for analysis. Daily counts of the emergency and hospital visits of heat-related illnesses (according to the 9th Revision of the International Classification of Diseases-Clinical Modification (ICD9-CM): 992 including heat stroke and heat exhaustion for 2002–2015 data, and ICD10-CM: T67.0XXA-T67.9XXA for 2016–2017 data) from the Health and Welfare Data Science Center, Ministry of Health and Welfare were used, comprising emergency/hospital visits of almost the entire population of Taiwan (23.55 million, mid-year of 2017). Data from May to October between 2002 and 2017 were included for analysis using SAS 9.4 (SAS Institute Inc., Cary, NC, USA) and R 3.5.1 with *mgcv* package. This heat-health evaluation was reviewed and approved by the Institutional Review Board of Academia Sinica (IRB No. AS-IRB-BM-18030). The authors had no access to information that could identify individual participants during or after data collection. The date of our analysis was from May 28^th^, 2018, to September 9^th^, 2021.

It should be emphasized that our method identified the points with “statistically significant” and “substantial” health impacts as thresholds rather than the points of minimal health risks obtained using typical epidemiological methods, which are often too low to be HHWS thresholds as pointed out by Cheng et al. [33] and Lung et al. [36]. For example, Taipei may issue summer-long warnings if the threshold is based on minimal risk. Details of the methodology can be found in Lung et al. [36] and are briefly described below.

Considering the two-day lags and avoiding interference of rare events, the final model used is as follows, with WBGT denoted as *D*_*t*_ and heat-related emergency or hospital visits denoted as *Y*_*t*_.

$$\log\left[E(Y_{t}|\theta )\right]=\alpha +\beta_{\theta 0}H_{t}+\beta_{\theta 1}H_{t-1}+\beta_{\theta 2}H_{t-2}+\gamma_{0}L_{t}+\gamma_{1}L_{t-1}+\gamma_{2}L_{t-2}+g(t)+\sum_{i=1}^{m}\lambda_{i}C_{it},$$ Eq (1)

$$where\;H_{t}=\left\{ \begin{array}{c} D_{t}-\theta ,\;if\;\delta >D_{t}>\theta \\ 0,\;\mathrm{otherwise} \end{array}\right.\;(the\;same\;definition\;for\;H_{t-1},\;H_{t-2})$$

$$and\;L_{t}=\left\{ \begin{array}{c} 1,\;\mathrm{if}\;D_{t}\geq \delta >\theta \\ 0,\;\mathrm{otherwise} \end{array}\right.\;(the\;same\;definition\;for\;L_{t-1},\;L_{t-2}).$$

where *θ* is a specified WBGT threshold candidate and *δ* is the chosen upper limit approximating the 99.5^th^ percentile of WBGT in the studied area (e.g., 36.5°C in the Taipei metropolitan area) with *L*_*t*_ as a dummy variable for removing the interference of rare events when WBGT exceeds *δ*. α is a constant, *g*(*t*) is a thin plate spline function for day *t*. Potential covariates on day *t*, denoted by *C*_*it*_, such as daily mean PM_2.5_ concentration (obtained from Taiwan Environmental Protection Administration), day of week, holiday, and periods prevalent with s*evere acute respiratory syndrome*, were considered for adjustment.

The threshold candidates were evaluated with the reference-adjusted risk ratio (RaRR) defined as:

$$RaRR=\frac{e^{{k\times \beta }_{\theta }}}{e^{{k\times \beta }_{a}}}=\frac{E(Y|{a,D}_{t}=b_{a}\leq a)}{E(Y|{\theta ,D}_{t}=b_{\theta }\leq \theta )}\times \frac{E(Y|{\theta ,D}_{t}=\theta +k)}{E(Y|{a,D}_{t}=a+k)}\;\mathrm{for}\;\theta \geq a.$$ Eq (2)

$e^{{k\times \beta }_{\theta }}$ is the risk ratio (RR), calculated as $E(Y_{t}|D_{t}=\theta +k)/E(Y_{t}|D_{t}=b_{\theta })$) for any value *b*_*θ*_≤*θ* and defined as the ratio of the expected health outcome for a *k* unit increase over the WBGT threshold candidate compared with the expected health outcome for any WBGT below or equal to the threshold. *a* is the lowest threshold candidate, and 30°C is the chosen reference. The RaRR is interpreted as the relative risk of the expected health outcome for exposure to the level of *k* units above the WBGT candidate compared with that for exposure to the same *k* units above the reference. The candidate *θ* with the maximum RaRR associated with statistically significant ${\hat{\beta }}_{\theta }$ is the selected threshold. $e^{\beta_{\theta j}/2}$ is used for calculating RaRR for a 0.5°C increase since the highest *θ* differs from *δ* only by 0.5°C sometimes.

### Risk communication concern

There were two major concerns regarding risk communication. The main concern was to evaluate whether the issuance of heat alerts was too frequent such that they would, on the one hand, cause the general public to become inattentive and indifferent, and, on the other hand, exhaust the resources of the authorities and private sectors responsible for responsive actions. Hence, the occurring frequency of WBGT exceeding the threshold candidates in the past 10 years was assessed. In view of the global warming trend, these past frequencies might be underestimates; yet, they provide a useful reference for anticipating the frequency of alert issuance in the coming years.

### Implementation of the TPE-pHHWS

The TPE-pHHWS was co-implemented by the science team and the TDEP in 2018. The warning threshold was determined by taking into account both scientific evidence and policy concerns. Of note is that instead of depending on the WBGT forecast, which the CWB could not provide at the time, the TPE-pHHWS issued warnings on the basis of the maximum hourly WBGT of the aforementioned four HOBO stations, and that heat alerts were only announced internally within the TDEP, and not to the public, for the purpose of triggering responsive actions. In 2015, the TDEP drafted the “Taipei City Heatwave Disaster Prevention and Rescue Countermeasures” and revised it over the years incorporating responsive programs devised by various city departments and heat-alert communication channels [45]. Implementation of the TPE-pHHWS offered an opportunity to refine those responsive programs and communication channels.

## Results and discussion

The scientific findings that address the three major policy concerns are presented, followed by implementation of the TPE-pHHWS. The discussion is associated with the results. The overall evaluation and lessons learnt from the co-design process are also discussed. The environmental data can be found in a data repository [46] while the health data cannot be shared due to privacy consideration.

### Temporal and spatial distribution of WBGT in Taipei City

The number of days from May to October in 2002–2016 (15-year historical trend) in Taipei City in the highest WBGT category of Japan and the US are presented in S2(A) and S2(B) Fig, respectively. There were at least 57 and 17 days every year, respectively, showing that Taipei residents were indeed exposed to WBGT levels of health concern. In 2016, there were even 124 and 101 days, respectively, with WBGT exceeding these health-concerning levels. Further analysis revealed that the 99th percentile of the daily WBGT maximum of warm seasons between 2002 and 2016 in Taipei were 36.8°C, with that between 2014 and 2016 reaching 37.4°C.

The spatial variation in WBGT was evaluated through HOBO monitoring in 2017 and 2018 and using TWIN data in 2018. Owing to administration delay, the HOBO monitoring in both years started in June. Hourly WBGT of 6 am–6 pm (daytime) obtained at HOBO and TWIN stations from June to October are summarized in Table 1. The highest HOBO-monitored WBGT (40.5°C) was observed at Station Central during the hours of 9am to noon in both years; this station also had the highest mean WBGT in all three time periods (6 am–6 pm, 9 am–noon, and noon–3 pm) as shown in Table 1. In the TWIN data, lower WBGT levels were obtained at altitude ≥ 100 m than at elevation < 100 m and HOBO stations (27.8–28.9°C in 2018) because the wind speeds are usually higher at higher altitude. The highest WBGT (42.1°C) occurred in one school at altitude < 100 m in 2018. The fact that these stations are situated on concrete surfaces, rather than grassland, explains why the observed WBGT levels are much higher than those expected from the historical trend (based on CWB data) shown above. Nevertheless, these WBGT values represent the heat stress levels that are actually experienced by residents.

**Table 1:** Hourly WBGT (6 am-6 pm) at four HOBO stations in June-October 2017 and 2018 and 57 TWIN stations in June-October 2018; paired t-test was used to compare the mean values during 9 am–noon versus noon–3 pm.

Stations	6 am–6 pm				≥ 32°C	≥ 34°C	≥ 36°C	9 am–noon				noon–3 pm			
	mean	SD	Max	N	(%)	(%)	(%)	mean	SD	Max	N	mean	SD	Max	N
**2017**															
**Station Central**	29.9	4.5	40.5	1835	34.5	20.8	9.5	32.5***	4.8	40.5	459	31.5	4.1	40.2	459
**Station East**	29.4	4.2	39.9	1835	28.8	13.7	3.9	31.4***	4.5	38.8	458	30.6	3.9	39.9	459
**Station South**	29.3	4.3	37.8	1835	30.6	14	2.4	31.4***	4.5	37.8	459	30.4	3.9	37.7	458
**Station West**	28.9	3.8	36.9	1834	22.2	5.3	0.5	30.6***	4	36.9	458	29.8	3.5	35.5	458
**2018**															
**Station Central**	28.9	4.5	40.5	1836	26.3	15	5.2	31.4***	4.6	40.5	459	30.2	4.2	40.2	459
**Station East**	28.5	4.3	38.8	1836	20.8	9.2	2.8	30.4***	4.5	38.4	459	29.5	4.1	38.8	459
**Station South**	28.3	4.3	38.1	1836	21.2	8.1	1	30.4***	4.4	38.1	459	29.3	3.9	36.5	459
**Station West**	27.8	3.8	36.2	1836	12.7	3.1	0.1	29.5***	4	36.2	459	28.5	3.5	35.6	459
**53 TWIN Stations with altitude < 100m**	28	4	42.1	99297	14.5	4	1.2								
**4 TWIN Stations with altitude ≥ 100m**	26.7	4.9	40.7	7030	15.1	6.7	2.7								

***: *p* < 0.001; Abbreviations: WBGT: wet-bulb globe temperature; TWIN: Taipei Weather Inquiry-based Learning Network; SD: standard deviation; MAX: maximum; N: number of observations

The heat-health impacts on the entire Taiwan population were found to be statistically significantly higher at WBGT ≥ 34°C [33, 36], while those for residents in the Taipei metropolitan area assessed in this study were statistically significantly higher at WBGT ≥ 36°C (please see the subsection “Heat-health impact evaluation”). Table 1 lists the percentages of WBGT exceeding 32, 34, and 36°C observed at both HOBO and TWIN stations. As can be seen, 0.5–9.5% and 0.1–5.2% of the observed WBGT exceeded 36°C in 2017 and 2018, respectively. In addition, 20.8% and 15% of the observed WBGT values exceeded 34°C in Station Central in 2017 and 2018, respectively. Taken together, these results demonstrate significant spatial variations in WBGT and indicate that severe heat stress was experienced by Taipei residents during the warm seasons.

**Fig 2:**
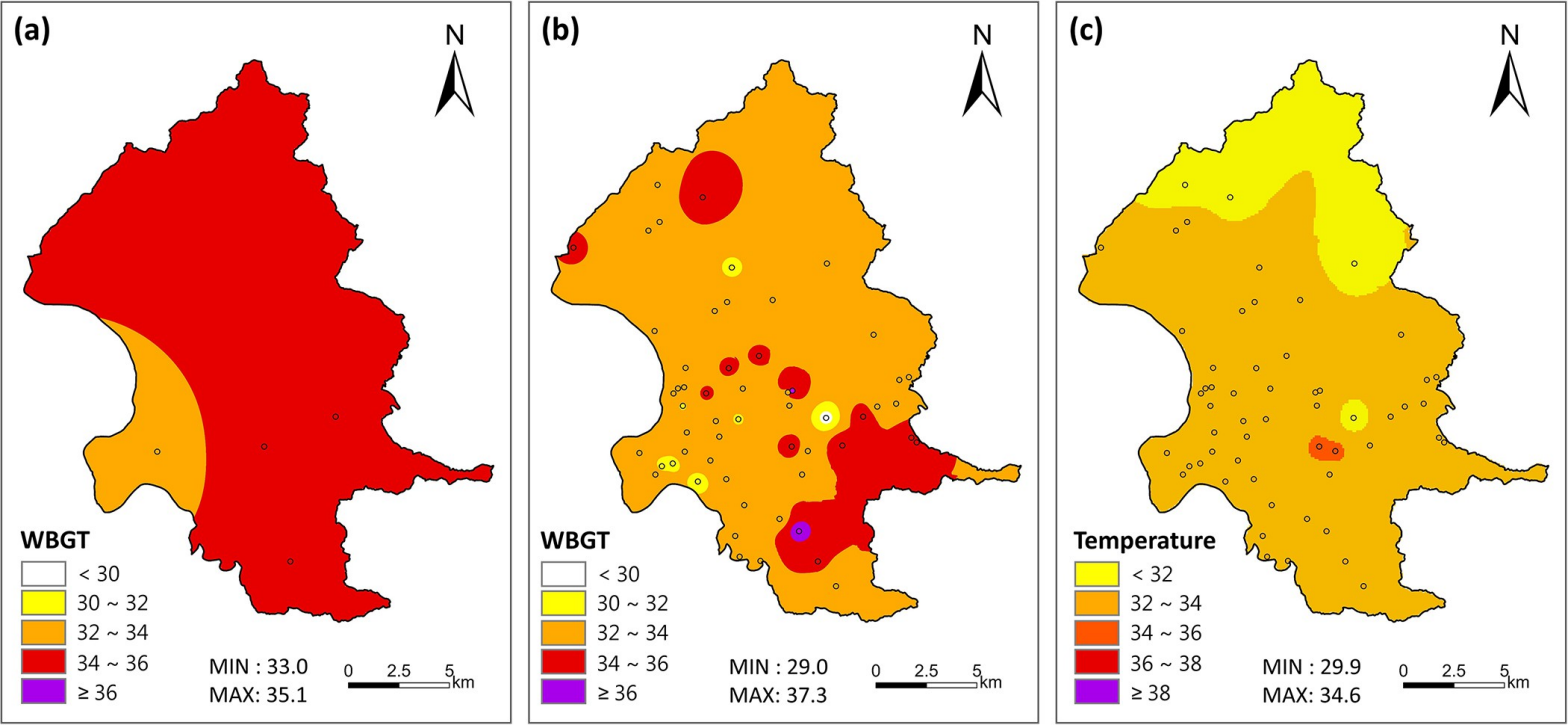
Spatial distribution of mean measurements between 9 and 11 am of August 11–17, 2018, obtained using inverse-distance-weighting interpolation of (a) WBGT from four HOBO stations, (b) WBGT from four HOBO and 52 TWIN stations, and (c) temperature from four HOBO and 52 TWIN stations. Of the total of 57 TWIN stations, 5 had high percentages of missing data during this period and were thus not included in the maps. Township and urban boundaries were obtained from open data [47] under a CC BY license, with permission from National Land Surveying and Mapping Center, Ministry of the Interior, Taiwan, original copyright 2015.

In addition, one case (between 9 am and 11 am of August 11–17, 2018) was presented to demonstrate spatial variations. With only observations of four HOBO stations, WBGT values in Taipei City were found in only two categories (32–34 and 34–36°C) (Fig 2(A)). Combining the observations of both HOBO and TWIN stations, much higher spatial resolutions of WBGT were found (Fig 2(B)); most areas had WBGT of 32–34°C and some had WBGT of 34–36°C, whereas two hot spots had WBGT ≥ 36°C.

While HOBO observations can be transmitted in real time and the calculated WBGT can be presented with a lead time of 10–15 minutes, TWIN and solar radiation data of CWB can only be obtained months afterwards. It would be advantageous for the TDEP to collaborate with the TDOE and all elementary schools to acquire real-time TWIN data for prompt issuance of heat warnings. However, TWIN is voluntarily maintained by each school; hence, both subsequent maintenance and data quality pose problems. For example, during the COVID-19 lockdown, when teachers and students were asked to teach/learn from home, the TWIN webpage was not properly maintained. Thus, this potential collaboration has challenges left unsolved. This demonstrates the difficulty of collaboration in a real-world situation where manpower, resource, and expertise are all potential limiting factors that restrain the establishment of a real-time HHWS.

Furthermore, the hot spots identified using WBGT would be missed out when temperature alone is the indicator. As shown in Fig 2(B) and 2(C), the two purple hot spots of WBGT were only in the orange category classified according to temperature. This discrepancy is hard evidence of the difference in results obtained using WBGT and temperature as the heat indicator. For people working or exercising outdoors, a WBGT-based HHWS provides actual hot spots of high heat stress to trigger appropriate self-protection actions that cannot otherwise be achieved when using indicators where solar radiation is not considered. Using inappropriate indicators may be one of the reasons for the ineffectiveness of the heat alerts in reducing mortality in US cities evaluated by Weinberger et al. [48]. While heat alerts with temperature and humidity may serve for their own purpose, e.g., for people staying indoors, adding additional indicators such as WBGT for people working or exercising outdoors could provide an extra layer of protection.

Moreover, our monitoring also pointed out that the hot periods in a diurnal cycle identified using WBGT as heat indicator differ from those identified using temperature, which usually reach the highest between noon and 2 pm. The WBGT values between 9 am and noon are statistically significantly higher than those between noon and 3 pm (Table 1). Taipei is a subtropical area with frequent afternoon showers in summertime. Clouds formed block solar radiation, resulting in lower WBGT between noon and 3 pm than between 9 am and noon, with less vigorous cloud formation. Using temperature as an indicator would mislead residents to work or exercise between 9 am and noon when they deem the “heat” indicated by temperature lower, while in reality, the “heat” is more severe between 9 am and noon as indicated by WBGT. This further demonstrates that (1) an effective HHWS requires appropriate indicators for identifying the actual high heat stress periods, and (2) local WBGT monitoring is important for revealing local features. These findings also have important implications for scheduling physical class for students to avoid heat exposure.

### Heat-health impact evaluation

The summary of 16-year data used for heat-health impact assessment is shown in Table 2. The mean values of daily WBGT maximum during warm seasons of these years are 30.1 ± 3.7°C. Daily emergency and hospital visits were 1.4 ± 2.1 and 80.3 ± 97.8, respectively. The majority of emergency and hospital visits were working-aged people (15–64 yrs).

**Table 2:** Summary of health and environment data for heat-health analysis (May to October, 2002–2017).

Variables	Mean	Standard Deviation	Minimum	Maximum
**Daily WBGT maximum (°C) (n = 2944)**	30.1	3.7	16.1	38.5
**Daily PM**_**2.5**_ **means (μg/m**^**3**^**) (n = 2919)**	22.1	11.3	2.1	111.2
**Daily emergency visits (n = 2944)**				
** Taipei metropolitan area**	1.4	2.1	0	22
** Female**	1.0	1.6	0	16
** Male**	0.4	0.9	0	12
** 0–14 yrs**	0.2	0.5	0	4
** 15–64 yrs**	1.0	1.7	0	16
** Above 65 yrs**	0.2	0.6	0	8
**Daily hospital visits (n = 2944)**				
** Taipei metropolitan area**	80.3	97.8	0	550
** Female**	32.2	38.6	0	235
** Male**	47.3	58.9	0	314
** 0–14 yrs**	2.7	3.2	0	23
** 15–64 yrs**	72.9	89.1	0	509
** Above 65 yrs**	4.7	6.6	0	54

Table 3(A) shows RRs at different WBGT threshold candidates on different lag days. RRs were increased with statistical significance for heat-related emergency and hospital visits when WBGT was 30°C. The highest RR at WBGT of 36°C on day 0 for emergency and hospital visits was 2.66 and 1.27, respectively. At WBGT of 36°C, RRs of emergency visits were statistically significant at lags 0 and 1 while those of hospital visits were statistically significant only at lag 0. Heat-health impacts typically have lag effects of 2–3 days [17]. For Taipei residents, statistically significant lag effects up to 2 days were observed when WBGT was 32 and 34 for emergency and hospital visits, respectively (Table 3(A)). RaRRs associated with WBGT of 36°C when RRs are statistically significant are presented in the last column. On day 0, the RaRRs for emergency and hospital visits were 2.42 and 1.22, respectively. Previous analysis for the whole of Taiwan found that the highest heat-health RaRR occurred at WBGT of 34°C [36]. The Taipei basin accumulates heat more easily than flat areas; thus, the identified WBGT threshold candidate is 36°C, higher than that for the whole of Taiwan (34°C).

**Table 3:** Risk ratios (RRs) at different WBGT threshold candidates for (a) different lag days for the entire population and (b) different sex and age groups on day 0, with the reference-adjusted risk ratios (RaRRs) associated with the highest threshold candidates of statistical significance.

**(a)**							
	**RR (95% confidence interval)**						**RaRR**
**Threshold**	**30°C**	**32°C**	**34°C**	**35°C**	**35.5°C**	**36°C**	
***Heat-related emergency visits***							
** Lag 0**	1.10*** (1.09, 1.12)	1.09*** (1.07, 1.11)	1.10*** (1.06, 1.14)	1.18*** (1.08, 1.29)	1.37*** (1.16, 1.62)	2.66*** **(1.70, 4.18)**	**2.42**
** Lag 1**	1.04*** (1.03, 1.06)	1.04*** (1.02, 1.06)	1.05* (1.01, 1.10)	1.07 (0.97, 1.18)	1.31** (1.08, 1.58)	2.34** **(1.35, 4.06)**	**2.25**
** Lag 2**	1.03*** (1.01, 1.04)	1.03*** (1.01, 1.05)	1.03 (0.99, 1.07)	1.04 (0.93, 1.16)	1.12 (0.90, 1.39)	0.78 (0.35, 1.74)	--
***Heat-related hospital visits***							
** Lag 0**	1.04*** (1.03,1.04)	1.03*** (1.02,1.03)	1.02*** (1.01,1.02)	1.00 (0.98,1.01)	0.97 (0.94,1.01)	1.27** **(1.10,1.46)**	**1.22**
** Lag 1**	1.02*** (1.02,1.02)	1.02*** (1.02,1.02)	1.02*** (1.01,1.02)	1.00 (0.99,1.01)	1.00 (0.97,1.03)	1.00 (0.91,1.09)	--
** Lag 2**	1.03*** (1.03,1.03)	1.03*** (1.02,1.03)	1.02*** (1.01,1.02)	1.00 (0.99,1.02)	1.00 (0.97,1.03)	1.03 (0.95,1.13)	--
**(b)**							
***Heat-related emergency visits***							
** Female**	1.07*** (1.04, 1.09)	1.05** (1.02, 1.08)	1.02 (0.95, 1.10)	0.95 (0.78, 1.15)	0.93 (0.63, 1.37)	1.48 (0.57, 3.87)	--
** Male**	1.12*** (1.10, 1.13)	1.11*** (1.08, 1.13)	1.13*** (1.08, 1.18)	1.27*** (1.15, 1.41)	1.56*** (1.29, 1.89)	3.46*** **(2.06, 5.81)**	**3.09**
** Age 0–14**	1.08*** (1.04, 1.12)	1.11*** (1.05, 1.17)	1.18* (1.04, 1.34)	1.44* (1.09, 1.91)	1.99** (1.19, 3.33)	8.32** **(1.96, 35.3)**	**7.70**
** Age 15–64**	1.11*** (1.09, 1.13)	1.09*** (1.07, 1.11)	1.10*** (1.05, 1.15)	1.16** (1.04, 1.29)	1.31** (1.07, 1.60)	2.52*** **(1.48, 4.30)**	**2.27**
** Age ≥ 65**	1.09*** (1.06, 1.13)	1.06** (1.02, 1.11)	1.07 (0.97, 1.17)	1.15 (0.93, 1.42)	1.30 (0.87, 1.93)	1.92 (0.67, 5.52)	--
***Heat-related hospital visits***							
** Female**	1.03*** (1.03,1.04)	1.02*** (1.02,1.03)	1.01*** (1.01,1.02)	0.99 (0.97,1.01)	0.95* (0.90,1.00)	1.21 (0.99,1.47)	--
** Male**	1.04*** (1.03,1.04)	1.03*** (1.02,1.03)	1.02*** (1.01,1.03)	1.01 (0.98,1.03)	1.00 (0.95,1.06)	1.36** **(1.10, 1.68)**	**1.31**
** Age 0–14**	1.04*** (1.04,1.05)	1.04*** (1.02,1.05)	1.03* (1.00,1.06)	1.05 (0.96,1.14)	1.12 (0.94,1.35)	2.11* **(1.13, 3.95)**	**2.03**
** Age 15–64**	1.04*** (1.03,1.04)	1.03*** (1.02,1.03)	1.02*** (1.01,1.02)	0.99 (0.98,1.01)	0.96 (0.93,1.00)	1.22* **(1.05, 1.43)**	**1.17**
** Age ≥ 65**	1.03*** (1.02,1.04)	1.02*** (1.01,1.03)	1.01 (0.99,1.04)	0.98 (0.92,1.04)	0.98 (0.85,1.12)	1.47 (0.88, 2.45)	--

*p < 0.05

** *p* < 0.01, and

*** *p* < 0.001

Table 3(B) shows the RRs at different WBGT threshold candidates for different sex and age groups on day 0, with details for different lags summarized in S1 Table. RaRRs for males at WBGT of 36°C were 3.09 and 1.31 for emergency and hospital visits, respectively, while those for females with insignificant RRs were not calculated. Among the different age groups, the RaRRs for those aged 0–14 were the highest, with 7.70 and 2.03 for emergency and hospital visits, respectively (Table 3(B)). Using an HHWS established with appropriate indicators, thresholds, and responsive actions, most of these heat-related emergency and hospital visits could be avoided. As extensively discussed in our earlier study [36], the threshold candidate with the highest RaRR is potentially the best one for an HHWS. According to Table 3(A) and 3(B), the threshold identified for significant heat-health impacts is 36°C.

### Frequency of days above thresholds

**Fig 3:**
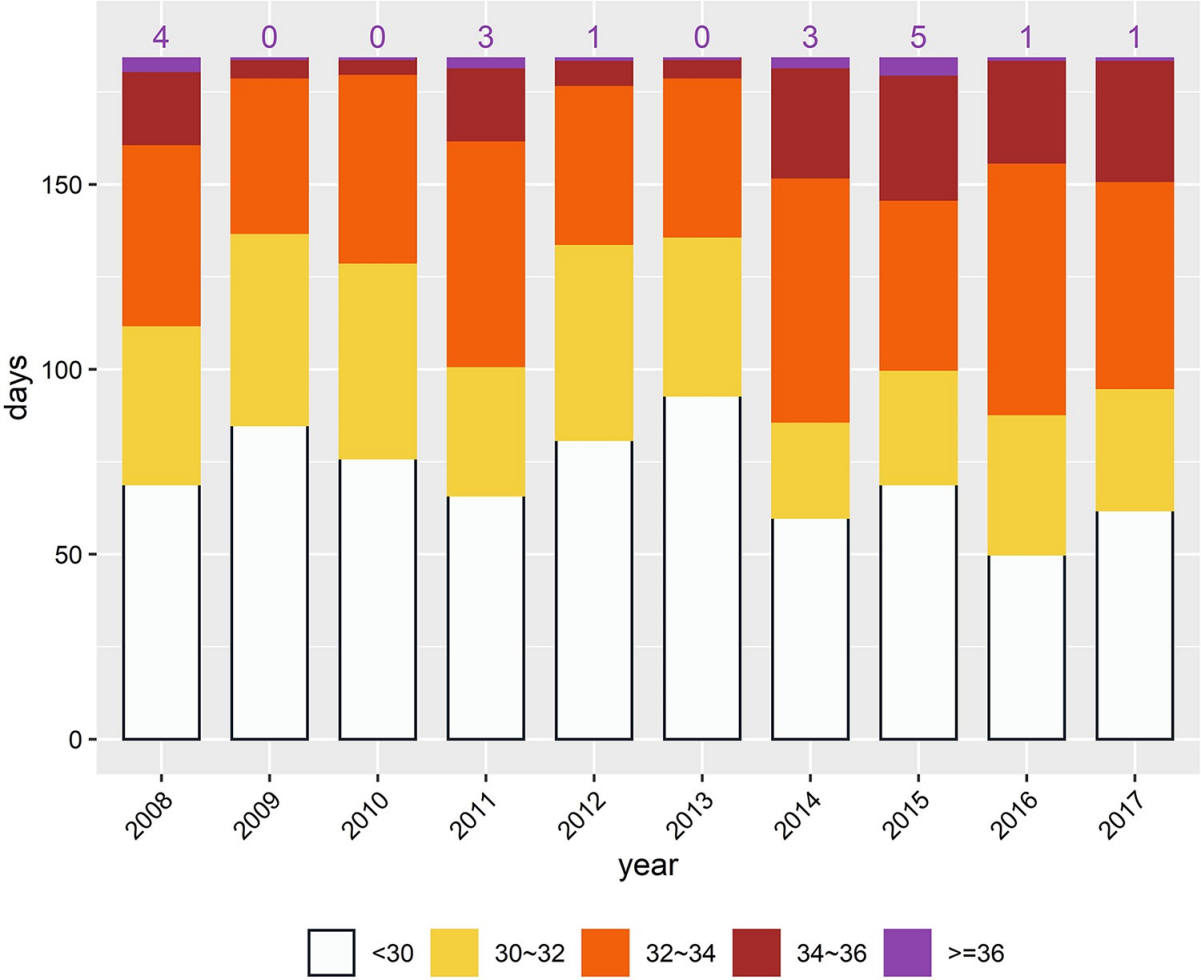
Number of days for different WBGT categories in May to October of 2008–2017. Number of days when WBGT ≥ 36°C are shown on the column top.

The frequencies for WBGT categories of < 30, 30–32, 32–34, 34–36, and ≥ 36°C over a 10-year period are presented in Fig 3. These five WBGT categories were chosen in view of the significant increase in heat-health risks for residents in the Taipei metropolitan area and the whole of Taiwan when WBGT exceeded 36°C and 34°C, respectively. As shown in the figure, between 2008 and 2017, the number of days with WBGT ≥ 36°C ranged from 0 to 5 days. The purpose of assessing the historical frequency of days with WBGT ≥ 36°C is to provide a ballpark estimate of the number of days when heat alerts are issued based on the threshold of 36°C. Since the criteria were only met on 0–5 days in the past ten years, selecting a WBGT threshold of 36°C may be suitable for Taipei City to avoid overly frequent announcements of heat warnings. Factoring into other considerations in different aspects, the WBGT threshold of this TPE-pHHWS was selected as described in the next subsection.

### Implementation of the TPE-pHHWS and planning of responsive actions

Integrating scientific evidence with policy concerns is a crucial step preceding system implementation. In summary, Taipei residents have indeed been exposed to health-concern heat stress levels during 2002–2018. Significant increases in heat-related emergency and hospital visits of Taipei residents were observed when WBGT exceeded 36°C with RaRRs of 2.42 and 1.22, respectively. The historical records from the CWB showed there were only 0 to 5 days with WBGT ≥ 36°C in 2008–2017, indicating infrequent occurrence. However, WBGT measured on concrete surfaces reflects the actual heat stress experienced by Taipei residents; thus, the frequency of WBGT ≥ 36°C observed at HOBO and TWIN stations was in fact much higher (Table 1); the exceedance percentage was up to 9.5% at one location. With WBGT forecast unavailable at the time, it was decided to use WBGT observations from the monitoring on concrete surfaces as a trigger of warning for the TPE-pHHWS. Therefore, it was speculated that the frequency of WBGT ≥ 36°C would be close to 5–10% of the days rather than only 0–5 days when based on CWB historical records obtained on grassland. After several round of discussion, it was agreed that the selected threshold for the TPE-pHHWS should be even higher than 36°C in order to avoid excessively frequent issuance of warnings. The consensus was to select a heat warning threshold of 36.8°C, which corresponds to the 99^th^ percentile of daily WBGT maximum of 2002–2016 in Taipei City. The TPE-pHHWS was implemented in 2018. There were in total 31 days in the summer 2018 with hourly maximum WBGT above 36.8°C. Among them, the maximum occurred 23 and 9 times in Station Central and East, respectively.

Furthermore, according to their own jurisdiction, ten departments and offices were involved in the formulation of responsive actions over the years (Table 4). For example, the Department of Environmental Protection increases the frequency of water-sprinkling on hot days in order to reduce the temperature, especially in the urban center with heat island effects. The Department of Labor promotes heat protection measures in outdoor workplaces and carries out inspections to ensure that proper measures are taken. The Department of Social Welfare sets up protective mechanisms for the elderly living alone and high-risk populations, and provides cooling equipment (e.g., electric fans) and temporary shelters with air-conditioning. The Department of Education adjusts class schedules to prevent students from engaging in outdoor activities under the heat. The Department of Health designs education brochures, strengthens emergency medical services, and formulates measures to reduce risk of food-borne diseases on hot days [45].

**Table 4:** Planning of responsive actions by different departments and offices of Taipei City Government [45].

Department or Office	Responsive actions
**Department of Environmental Protection**	Sprinkling water on hot days
**Fire Department**	Responding to heat-related disasters and offering rescue
**Department of Health**	Devising health education information and promoting food safety
**Department of Social Welfare**	Taking care of vulnerable groups and providing temporary shelters to reduce heat-health risks
**Department of Labor**	Conducting inspections at outdoor workplaces and promoting protection of workers’ health
**Department of Education**	Designing education materials and adjusting schedules for outdoor classes
**Park and Street Lights Office**	Watering plants and assisting with cooling
**Market Administration Office**	Conducting market inspection for food safety
**Hydraulic Engineering Office**	Removing dead fish from rivers/streams on hot days
**Sewerage Systems Office**	Managing and controlling sewerage closure and release

In addition, the chain of command was designed according to disaster-response procedures. In 2018, within the Taipei City Government, heat-health warnings were designed to be issued by either of two mechanisms. One was the WBGT forecast issued by the CWB, which may be available in the future, and the other was WBGT monitoring conducted by the TDEP through four HOBO weather stations. When a warning is issued, the TDEP first informs the Fire Department, which responds to all types of disaster. The Fire Department then notifies the relevant departments to initiate responsive measures through the short messaging service system [45]. This chain of command was put into practice when the TPE-pHHWS was implemented.

During implementation of the TPE-pHHWS, the CWB unexpectedly provided “heat information” to the public starting from June 15, 2018, using temperature as the indicator. Although CWB officials were convinced that WBGT was the preferred choice, forecasting of WBGT was a major challenge yet to resolve. For consistency among government agencies, the TDEP decided to adopt “heat information” as the only trigger mechanism for responsive actions after 2018. Responsive actions are initiated when the daily maximum temperature reaches 36°C for three consecutive days or when daily maximum temperature reaches 38°C (S3(A) Fig).

At first glance, the TDEP’s change in taking temperature instead of WBGT as a reference for triggering responsive actions would imply failure of the TPE-pHHWS. Nevertheless, the interaction during the co-design process did assist government officials in identifying the scientific evidence needed for establishing an HHWS. In addition, the proactive attitude of the TDEP in implementing the TPE-pHHWS did put extra pressure on the CWB to provide “heat information”. Thus, the TPE-pHHWS did pave the way for these advancements. Moreover, the interactions of our science team with officials of the TDEP and CWB continued. Consistent relationships of WBGT with heat-related morbidity [33, 36] and different hot spots and periods identified using WBGT, as demonstrated in Tables 1, 3 and Fig 2, further compel CWB to adopt WBGT as the indicator for heat alerts to be issued for health risk reduction. After several years of evaluation, the CWB started to provide daily forecast of WBGT in one of their specialized cellphone apps in 2021 and, eventually, in their main cellphone app in 2022. As a result, the TDEP decided to adopt the WBGT forecast as an additional trigger of responsive actions in 2022. In other words, the chain of command designed in 2018 was modified as “triggering responsive actions within the Taipei City Government according to the CWB forecast when (1) daily maximum temperature reaches 36°C for three consecutive days or daily maximum temperature reaches 38°C (S3(A) Fig) or (2) WBGT exceeds 36°C (S3(B) Fig)”. The decision of selecting WBGT of 36°C as a warning threshold was made by CWB based on two reasons. First, one-third of Taiwanese live in the Taipei metropolitan area, and epidemiological evidence showed that the RRs of the heat-related emergency and hospital visits in this area were significantly increased at WBGT of 36°C, as shown in Table 3. Secondly, it is easier to communicate with the general public regarding a threshold value of an integer such as 36°C rather than a number with decimal places such as 36.8°C. Again, CWB’s consideration includes biometeorological, epidemiological and public health, and risk communication aspects. In summary, with some twists and turns, the scientific findings that formed the basis of the TPE-pHHWS eventually accomplished their mission and assisted in developing a nationwide heat-alert app.

### Overall evaluation

HHWS is an important health adaptation strategy and the co-design process is viewed as a critical element in transdisciplinary research which emphasizes facilitating societal transformation toward sustainability [3, 9, 20]. To our best knowledge, this is the first publication describing the interactive co-design process of establishing a city-level pilot HHWS. This paper fills the research gap and describes the required scientific evidence as deliberated at the co-design meetings in light of the three aspects of concern emphasized in the Guidance on Warning-System Development [25]. It was essential to frame the research questions together with the key stakeholders in the beginning of this study such that the acquired data and knowledge could be directly fed into decision making. Afterwards, a city-level pilot HHWS was co-implemented in 2018, and accompanying responsive actions were formulated and carried out. The scientific findings in carrying out this TPE-pHHWS provide key evidence for establishing a nationwide heat-alert app. The present case serves as an example in transdisciplinary research for the translation of knowledge into action toward tackling challenges associated with meeting the SDGs. In particular, for low- and middle-income countries, a city-level pilot HHWS is a cost-effective alternative preceding the establishment of a nationwide HHWS.

In the beginning of the co-design process, selecting an appropriate indicator is considered as an essential step for establishing an effective HHWS. WBGT was selected as the indicator in this TPE-pHHWS. Typically, the inertia within the governmental system serves as hindrance for adopting any new indicators. The WMO has emphasized climate service in the past 15 years [49]. Under the threat of climate change, policy makers are under pressure to provide new services for public health protection. This represents a window of opportunity for scientists collaborating with policy makers to establish an expanded or modified HHWS with evidence-based heat indicators, such as WBGT, in order to reduce heat-health risks, especially for highly exposed outdoor workers or those who exercise or run errands outdoors. The TPE-pHHWS described in this paper is a successful demonstration.

Our experience revealed that policy makers at the city level are more willing to make changes than those in central government. That less resources are required and the sole leadership at the city level are two advantages that contribute to breaking institutional inertia and fostering changes. In this TPE-pHHWS, the TDEP received the support of the mayor and organized a series of meetings within the city government to move forward with formulating responsive actions to be carried out by various departments. Strong support from the mayor and city officials was the vital force behind the successful implementation of this pilot HHWS.

Of the three major policy concerns, the most challenging one is to provide threshold candidates for selection on the basis of local health records, because traditional epidemiological methods are not designed for this. Thus, the statistical method was modified to obtain results for meeting this policy concern. In addition, we used morbidity evidence instead of mortality records, as adopted by other HHWSs, because the purpose of establishing the HHWS is to prevent heat-related morbidity. Many countries may not have a nationwide morbidity database. Nevertheless, the consideration behind the threshold selection could still provide important insights to scientists and policy makers in other countries.

Toloo et al. [50] conducted a systematic review of 15 studies and evaluated the effectiveness of the developed HHWS; their conclusion was that due to the presence of other contributory factors, the effectiveness of such systems in reducing mortality and morbidity requires further investigation. Additionally, Weinberger et al. [48] evaluated the effectiveness of heat alerts issued by the National Weather Service in 20 US cities from 2001 to 2006 and found no association between heat alerts and lower mortality in most of the studied cities. Nevertheless, they emphasized the importance of the accompanying intervention strategies in reducing heat-related mortality. In our case, city officials made a lot of efforts in formulating responsive actions. Additionally, conducting monitoring to reveal the local features of heat indicators and select warning thresholds based on local health evidence should be taken as prerequisites for establishing an effective HHWS.

In several recent publications, considerable spatial variations in heat-health risks were found during heatwaves in the UK, Australia, and Japan, with emphasis that the weather agencies should consider regional differences when issuing HHWS warnings or formulating responsive actions [12–14, 38]. In Taiwan, even though there is spatial variation in heat-health risks [36], establishing a nationwide unified HHWS is deemed as the first step. The effectiveness of this HHWS should be evaluated later in order to determine whether a HHWS based on finer spatial resolution is needed.

In a book containing case studies involving co-designed transdisciplinary approaches with research on mobile health check-up, disaster prevention, land use, and community development [7], the importance for next-generation scientists who will tackle social issues to gain co-design experience is emphasized. The current paper provides another case with co-designed transdisciplinary approaches focusing on HHWS establishment, which is urgently needed around the world. It serves to promote more co-designed research works to fulfill societal needs.

There are certain limitations of the TPE-pHHWS. Firstly, WBGT monitoring was not conducted on grassland, as required by WMO, making it difficult for comparison with other WBGT values derived from meteorological parameters taken at standard CWB stations. We acknowledge that unified standard operational procedures are needed for comparison with international weather stations worldwide. However, most people in Taiwan do not live on grassland; hence, the WBGT obtained from stations situated on grassland does not represent the actual heat stress exposure experienced by the residents. Such discrepancy in WBGT monitoring cannot be eliminated due to the different purposes of measurement and our insistence on assessing the actual heat stress for morbidity prevention. Secondly, the threshold used for the TPE-pHHWS (36.8°C) was not selected according to the scientific findings of WBGT–health evaluation (36°C) for the reasons described above. In this instance, the policy considerations outweighed the scientific evidence; our scientific team also agreed that overly frequent warnings should be avoided. In reality, 36.8°C does not differ much from 36.0°C. Furthermore, compromises are inevitable once scientists become involved in the co-design and co-implementation process, and different preferences and perspectives need to be reconciled. As a matter of fact, even though 36.8°C was chosen in the TPE-pHHWS implemented in 2018, 36°C was eventually selected by the CWB as the threshold for issuing nationwide heat-health warnings in their cellphone app in 2021. Thus, the heat-health evaluation conducted in this study contributes its share after all.

## Conclusions

Transforming knowledge into action is necessary when tackling real-world issues, such as in reducing health risks under a climate emergency. Co-design is crucial in facilitating the integration of scientific knowledge and policy considerations in order to formulate health adaptation strategies. This paper showcases a pilot HHWS developed for Taipei, Taiwan, based on a co-design process wherein research gaps were identified regarding three aspects (biometeorology, epidemiology and public health, and risk communication) that were emphasized by the WMO and WHO. The scientific findings addressing policy concerns are also presented. Taipei residents have indeed been exposed to health-concerning heat stress levels during 2002–2018. A significant increase in heat-related emergency and hospital visits of Taipei residents was observed when WBGT exceeded 36°C. Moreover, the fact that hot spots and periods identified using WBGT were quite different from those identified using temperature further highlights the importance of adopting the appropriate heat stress indicator in the HHWS to reduce health risks. It was also shown that local monitoring of WBGT is essential to reveal the local features of residents’ heat exposure, and thresholds for heat-health warning should be selected according to the local health evidence. The scientific evidence acquired for this TPE-pHHWS served as a useful reference for developing a nationwide heat-alert app. The lessons learnt can provide important insights to scientists and policy makers in setting up an HHWS in their own city/country and for scientists interested in taking on transdisciplinary research to tackle other sustainability challenges.

## Supporting information

S1 Table Risk ratios (RRs) of different WBGT threshold candidates for heat-related (a) emergency and (b) hospital visits on different lag days of different sex and age groups with the reference-adjusted risk ratios (RaRRs) associated with the highest threshold candidates of statistical significance. RR and RaRRs for the entire Taipei population are listed in the first three rows for comparison.
(PDF)

S1 Fig Monitoring stations in Taipei City. The blue and black lines show the border of Taipei City and the entire Taipei metropolitan area, respectively; the magenta and purple dots are 53 and 4 stations in elementary schools at altitude < and ≥ 100 m, respectively; the star signs indicate the four HOBO monitoring stations of the TPE-pHHWS. Grid numerical terrain model data (20-meter resolution) were obtained from open data [https://data.gov.tw/dataset/35430] under a CC BY license, with permission from Department of Land Administration, Ministry of the Interior, Taiwan, original copyright 2016. Township and urban boundaries were obtained from open data [https://data.gov.tw/dataset/7441] under a CC BY license, with permission from National Land Surveying and Mapping Center, Ministry of the Interior, Taiwan, original copyright 2015.
(TIF)

S2 Fig Number of days above thresholds for (a) Japan WBGT categories for the general public and (b) US WBGT categories for the workers. Number of days in the highest WBGT category are listed on the top of the respective column.
(TIF)

S3 Fig (a) Classification of temperature according to “heat information” announced by Central Weather Bureau (https://www.cwb.gov.tw/V8/E/P/Warning/W29.html (English)) and (b) the classification of Wet-bulb Globe Temperature (WBGT) translated from the data description of https://opendata.cwb.gov.tw/dataset/forecast/M-A0085-001 in Mandarin posted by Central Weather Bureau. Note: The Central Weather Bureau has been upgraded to the Central Weather Administration on September 15, 2023; thus, the webpage may be renamed as www.cwa.gov.tw in the future.
(TIF)

## Abbreviations

CWB: Central Weather Bureau
HHWS: heat-health warning system
ICD: international classification of disease
PM_2.5_: particulate matter with aerodynamic diameter less than or equal to 2.5 μm
RaRR: reference-adjusted risk ratio
RR: risk ratio
SDG: sustainable development goal
TDOE: Department of Education of Taipei City Government
TDEP: Department of Environmental Protection of Taipei City Government
TWIN: Taipei Weather Inquiry-based Learning Network
WBGT: wet-bulb globe temperature
WHO: World Health Organization
WMO: World Meteorological Organization

## References

[1] National Aeronautics and Space Administration (NASA). A July of Extremes—NASA Earth Observatory, 2022; Available from: https://earthobservatory.nasa.gov/images/150152/a-july-of-extremes

[2] Intergovernmental Panel on Climate Change (IPCC). Climate Change 2021: The physical science basis. In: Masson-Delmotte VP, Zhai A, editors. Summary for policymakers. Contribution of working group I to the Sixth assessment report of the intergovernmental panel on climate change; 2021. pp. 3−32.

[3] Lang DJ, Wiek A, Bergmann M, Stauffacher M, Martens P, Moll P, et al. Transdisciplinary research in sustainability science: practice, principles, and challenges. Sustain Sci. 2012;7(S1): 25–43. doi: 10.1007/s11625-011-0149-x

[4] Future Earth. Future Earth Strategic Research Agenda 2014. Paris: International Council for Science (ICSU). 2014; Available from: https://futureearth.org/2014/12/04/future-earth-strategic-research-agenda-2014-published/

[5] Future Earth. Our History, Future Earth. 2022; Available from: https://futureearth.org/about/history

[6] van der Hel S. New science for global sustainability? The institutionalisation of knowledge co-production in Future Earth. Environ Sci Policy. 2016;61: 165–75. doi: 10.1016/j.envsci.2016.03.012

[7] Yahara T. Decision science for Future Earth: Theory and practice. Singapore: Springer Singapore Pte. Limited; 2021.

[8] United Nation Environmental Programme (UNEP). Facts about the climate emergency. 2020; Available from: https://www.unenvironment.org/explore-topics/climate-change/facts-about-climate-emergency

[9] Mauser W, Klepper G, Rice M, Schmalzbauer BS, Hackmann H, Leemans R, et al. Transdisciplinary global change research: the co-creation of knowledge for sustainability. Curr Opin Environ Sustain. 2013;5(3–4): 420–31. doi: 10.1016/j.cosust.2013.07.001

[10] Watts N, Adger WN, Ayeb-Karlsson S, Bai Y, Byass P, Campbell-Lendrum D, et al. The lancet countdown: Tracking progress on health and climate change. Lancet. 2017;389(10074): 1151–64. doi: 10.1016/S0140-6736(16)32124-9 27856085

[11] World Meteorological Organization (WMO). WMO Provisional Report on the State of the Global Climate 2020. 2020; Available from: https://library.wmo.int/index.php?lvl=notice_display&id=21804#.ZBpkVnZBxPY

[12] Thompson R, Landeg O, Kar-Purkayastha I, Hajat S, Kovats S, O’Connell E. Heatwave mortality in summer 2020 in England: An observational study. Int J Environ Res Public Health. 2022;19(10). doi: 10.3390/ijerph19106123 35627660 PMC9141696

[13] Franklin RC, Mason HM, King JC, Peden AE, Nairn J, Miller L, et al. Heatwaves and mortality in Queensland 2010–2019: implications for a homogenous state-wide approach. Int J Biometeorol. 2023;67(3): 503–15. doi: 10.1007/s00484-023-02430-6 36735072 PMC9974727

[14] Oka K, Honda Y, Hijioka Y. Launching criteria of ‘Heatstroke Alert’ in Japan according to regionality and age group. Environ Res Commun. 2023;5(2). doi: 10.1088/2515-7620/acac03

[15] Yan M, Xie Y, Zhu H, Ban J, Gong J, Li T. Cardiovascular mortality risks during the 2017 exceptional heatwaves in China. Environ Int. 2023;172: 107767. doi: 10.1016/j.envint.2023.107767 36716635

[16] Sharma A, Andhikaputra G, Wang YC. Heatwaves in South Asia: Characterization, consequences on human health, and adaptation strategies. Atmosphere. 2022;13(5). doi: 10.3390/atmos13050734

[17] Lo YTE, Mitchell DM, Thompson R, O’Connell E, Gasparrini A. Estimating heat-related mortality in near real time for national heatwave plans. Environ Res Lett. 2022;17(2): 024017. doi: 10.1088/1748-9326/ac4cf4 35341022 PMC7612535

[18] Burgstall A, Casanueva A, Kotlarski S, Schwierz C. Heat warnings in Switzerland: Reassessing the choice of the current heat stress index. Int J Environ Res Public Health. 2019;16(15): 2684. doi: 10.3390/ijerph16152684 31357581 PMC6696474

[19] Casanueva A, Burgstall A, Kotlarski S, Messeri A, Morabito M, Flouris AD, et al. Overview of existing heat-health warning systems in Europe. Int J Environ Res Public Health. 2019;16(15): 2657. doi: 10.3390/ijerph16152657 31349585 PMC6695887

[20] National Weather Service (NWS). National Oceanic and Atmospheric Administration (NOAA). Heat Index. 2021; Available from: https://www.weather.gov/safety/heat-index

[21] Government of Canada. Warm season weather hazards. 2019; https://www.canada.ca/en/environment-climate-change/services/seasonal-weather-hazards/warm-season-weather-hazards.html

[22] Australian Government—Bureau of Meteorology. Homepage: Heatwave Service for Australia. 2018; Available from: http://www.bom.gov.au/australia/heatwave/index.shtml

[23] Japanese Ministry of the Environment. Homepage: Heat Illness Prevention Information. 2018; Available from: http://www.wbgt.env.go.jp/en/

[24] Lee KL, Chan YH, Lee TC, Goggins WB, Chan EYY. The development of the Hong Kong Heat Index for enhancing the heat stress information service of the Hong Kong Observatory. Int J Biometeorol. 2016;60(7): 1029–39. doi: 10.1007/s00484-015-1094-7 26546311

[25] World Meteorological Organization (WMO) and World Health Organization (WHO). Heatwaves and health: Guidance on warning-system development. WMO-No. 1142, World Meteorological Organization, Geneva, Switzerland. 2015.

[26] Broede P, Fiala D, Lemke B, Kjellstrom T. Estimated work ability in warm outdoor environments depends on the chosen heat stress assessment metric. Int J Biometeorol. 2018;62(3): 331–45. doi: 10.1007/s00484-017-1346-9 28424950

[27] Błażejczyk K, Baranowski J, Błażejczyk A. Heat stress and occupational health and safety–spatial and temporal differentiation. Misc Geogr. 2014;18(1): 61–7. doi: 10.2478/mgrsd-2014-0011

[28] Hyatt OM, Lemke B, Kjellstrom T. Regional maps of occupational heat exposure: past, present, and potential future. Glob Health Action. 2010;3: 10. doi: 10.3402/gha.v3i0.5715 21165172 PMC3002252

[29] ISO (International Standards Organization). Ergonomics of the Thermal Environment—Assessment of Heat Stress Using the WBGT (Wet Bulb Globe Temperature) Index. 2017; Available from: https://www.iso.org/standard/67188.html

[30] Spector JT, Sheffield PE. Re-evaluating Occupational Heat Stress in a Changing Climate. Ann Occup Hyg. 2014;58(8): 936–42. doi: 10.1093/annhyg/meu073 25261455 PMC4481564

[31] Yaglou CP, Minard D. Prevention of heat casualties at marine corps training centers. Armed Services Technical Information Agency Document Service Center AD099920. 1956; Available from: https://apps.dtic.mil/sti/tr/pdf/AD0099920.pdf

[32] Bernard TE. Thermal stress. In: Fundamentals of industrial hygene edition 6^th^. National Safety Council, 6th Edition; 2012. pp. 26–28.

[33] Cheng YT, Lung SCC, Hwang JS. New approach to identifying proper thresholds for a heat warning system using health risk increments. Environ Res. 2019;170: 282–92. doi: 10.1016/j.envres.2018.12.059 30599292 PMC7126132

[34] Kakamu T, Wada K, Smith DR, Endo S, Fukushima T. Preventing heat illness in the anticipated hot climate of the Tokyo 2020 Summer Olympic Games. Environ Health Prev. 2017;22(1). doi: 10.1186/s12199-017-0675-y 29165162 PMC5664809

[35] Lin YK, Chang CK, Li MH, Wu YC, Wang YC. High-temperature indices associated with mortality and outpatient visits: Characterizing the association with elevated temperature. Sci Total Environ. 2012;427: 41–9. doi: 10.1016/j.scitotenv.2012.04.039 22575378 PMC7127034

[36] Lung SCC, Yeh JCJ, Hwang JS. Selecting thresholds of heat-warning systems with substantial enhancement of essential population health outcomes for facilitating implementation. Int J Environ Res Public Health. 2021;18(18): 9506. doi: 10.3390/ijerph18189506 34574429 PMC8471601

[37] Nakamura D, Kinoshita H, Asada K, Arimitsu T, Yasumatsu M, Ishiwata T. Trends in ambulance dispatches related to heat illness from 2010 to 2019: An ecological study. PLoS One. 2022;17(11): e0275641. doi: 10.1371/journal.pone.0275641 36342929 PMC9639828

[38] Ueno S, Hayano D, Noguchi E, Aruga T. Investigating age and regional effects on the relation between the incidence of heat-related ambulance transport and daily maximum temperature or WBGT. Environ Health Prev Med. 2021;26(1): 116. doi: 10.1186/s12199-021-01034-z 34893022 PMC8903699

[39] Coccolo S, Kampf J, Scartezzini JL, Pearlmutter D. Outdoor human comfort and thermal stress: A comprehensive review on models and standards. Urban Clim. 2016;18: 33–57. doi: 10.1016/j.uclim.2016.08.004

[40] Toloo G, FitzGerald G, Aitken P, Verrall K, Tong S. Evaluating the effectiveness of heat warning systems: systematic review of epidemiological evidence. Int J Environ Res Public Health. 2013;58(5): 667–81. doi: 10.1007/s00038-013-0465-2 23564031

[41] United Nations (UN). Sustainable Development Goals (SDGs). 2018; Available from: https://www.un.org/sustainabledevelopment/

[42] Honda Y, Kondo M, McGregor G, Kim H, Guo YL, Hijioka Y, et al. Heat-related mortality risk model for climate change impact projection. Environ Health Prev. 2014;19(1): 56–63. doi: 10.1007/s12199-013-0354-6 23928946 PMC3890078

[43] Liljegren JC, Carhart RA, Lawday P, Tschopp S, Sharp R. Modeling the wet bulb globe temperature using standard meteorological measurements. J Occup Environ Hyg. 2008;5(10): 645–55. doi: 10.1080/15459620802310770 18668404

[44] Air Force USA. Thermal injury prevention program. Air Force Instruction 48–151. 2016; Available from: www.e-Publishing.af.mil

[45] Taipei Department of Environmental Protection (TDEP). Taipei City Heatwave Warning Notification and Response Procedure. 2022; Available from: https://english.dep.gov.taipei/News_Content.aspx?n=E56771B61602F277&sms=DFFA119D1FD5602C&s=3DFE7B7601ED68AB

[46] Lung SCC. Environmental data in Taipei during 2002–2020; 2023 [cited 2023 Jun 20]. Database: depositar [Internet]. Available from: https://data.depositar.io/zh_Hant_TW/dataset/environment_data_in_taipei_during_2002-2020

[47] National Land Surveying and Mapping Center, Open Data on Township and urban boundaries (TWD97 latitude and longitude), National Land Surveying and Mapping Center, Ministry of the Interior, original copyright 2015, https://data.gov.tw/dataset/7441.

[48] Weinberger KR, Zanobetti A, Schwartz J, Wellenius GA. Effectiveness of National Weather Service heat alerts in preventing mortality in 20 US cities. Environ Int. 2018;116: 30–8. doi: 10.1016/j.envint.2018.03.028 29649774 PMC5970988

[49] Tall A. What do we mean by climate services? World Meteorological Organization (WMO). Bulletin n°: Vol 62 (Special Issue). 2013; Available from: https://public.wmo.int/en/bulletin/what-do-we-mean-climate-services

[50] Toloo G, Fitzgerald G, Aitken P, Verrall K, Tong S. Are heat warning systems effective? Environ Health. 2013;12(1): 27. doi: 10.1186/1476-069X-12-27 23561265 PMC3626939

