# Supplementary material for: A pilot heat-health warning system co-designed for a subtropical city
Source: PLoS One. 2023 Nov 10;18(11):e0294281. doi: 10.1371/journal.pone.0294281 (PMC10637700; doi:10.1371/journal.pone.0294281)
Supplement: S1 Table — RR and RaRRs for the entire Taipei population are listed in the first three rows for comparison. (PDF) [file pone.0294281.s001.pdf]

S1 Table Risk ratios (RRs) at different WBGT threshold candidates for heat-related (a) emergency and (b) hospital visits on different lag days of different sex and age groups with the reference-adjusted risk ratios (RaRRs) associated with the highest threshold candidates of statistical significance; RR and RaRRs for the entire Taipei population are listed in the first three rows for comparison

	**RR (95% confidence interval)**						**RaRR**
**Threshold**	**30°C**	**32°C**	**34°C**	**35°C**	**35.5°C**	**36°C**	
(a) <u>Heat-related emergency visits</u>							
Lag0	1.10*** (1.09, 1.12)	1.09*** (1.07, 1.11)	1.10*** (1.06, 1.14)	1.18*** (1.08, 1.29)	1.37*** (1.16, 1.62)	**2.66*** (1.70, 4.18)**	**2.42**
Lag1	1.04*** (1.03, 1.06)	1.04*** (1.02, 1.06)	1.05* (1.01, 1.10)	1.07 (0.97, 1.18)	1.31** (1.08, 1.58)	**2.34** (1.35, 4.06)**	**2.25**
Lag2	1.03*** (1.01, 1.04)	1.03*** (1.01, 1.05)	1.03 (0.99, 1.07)	1.04 (0.93, 1.16)	1.12 (0.90, 1.39)	0.78 (0.35, 1.74)	
<u>Female</u>							
Lag0	1.07*** (1.04, 1.09)	1.05** (1.02, 1.08)	1.02 (0.95, 1.10)	0.95 (0.78, 1.15)	0.93 (0.63, 1.37)	1.48 (0.57, 3.87)	
Lag1	1.06*** (1.03, 1.08)	1.06*** (1.03, 1.10)	1.06 (0.99, 1.15)	1.09 (0.90, 1.33)	1.34 (0.93, 1.94)	2.64 (0.95, 7.31)	
Lag2	1.03* (1.01, 1.05)	1.03 (1.00, 1.07)	1.03 (0.95, 1.11)	1.01 (0.81, 1.24)	1.09 (0.70, 1.70)	1.90 (0.52, 6.91)	

	RR (95% confidence interval)						RaRR
**Threshold**	**30°C**	**32°C**	**34°C**	**35°C**	**35.5°C**	**36°C**	
Male							
Lag0	1.12***<br>(1.10, 1.13)	1.11***<br>(1.08, 1.13)	1.13***<br>(1.08, 1.18)	1.27***<br>(1.15, 1.41)	1.56***<br>(1.29, 1.89)	**3.46***<br>(2.06, 5.81)**	**3.09**
Lag1	1.04***<br>(1.03, 1.06)	1.04***<br>(1.02, 1.06)	1.06*<br>(1.01, 1.11)	1.08<br>(0.96, 1.22)	1.34*<br>(1.07, 1.68)	**2.33*<br>(1.21, 4.50)**	**2.24**
Lag2	1.02**<br>(1.01, 1.04)	1.03**<br>(1.01, 1.05)	1.03<br>(0.98, 1.08)	1.03<br>(0.91, 1.17)	1.09<br>(0.84, 1.41)	0.49<br>(0.17, 1.44)	
Age 0-14							
Lag0	1.08***<br>(1.04, 1.12)	1.11***<br>(1.05, 1.17)	1.18*<br>(1.04, 1.34)	1.44*<br>(1.09, 1.91)	1.99**<br>(1.19, 3.33)	**8.32**<br>(1.96, 35.3)**	**7.70**
Lag1	1.00<br>(0.96, 1.04)	0.98<br>(0.92, 1.05)	0.95<br>(0.80, 1.11)	0.77<br>(0.48, 1.24)	0.68<br>(0.25, 1.88)	0.37<br>(0.01, 10.5)	
Lag2	1.00<br>(0.97, 1.04)	1.01<br>(0.95, 1.07)	1.10<br>(0.95, 1.27)	1.32<br>(0.96, 1.83)	1.36<br>(0.66, 2.79)	0.01<br>(0.00,258.9)	
Age 15-64							
Lag0	1.11***<br>(1.09, 1.13)	1.09***<br>(1.07, 1.11)	1.10***<br>(1.05, 1.15)	1.16**<br>(1.04, 1.29)	1.31**<br>(1.07, 1.60)	**2.52***<br>(1.48, 4.30)**	**2.27**
Lag1	1.05***<br>(1.03, 1.07)	1.04***<br>(1.02, 1.07)	1.05*<br>(1.00, 1.10)	1.07<br>(0.95, 1.21)	1.30*<br>(1.04, 1.62)	**2.12*<br>(1.10, 4.09)**	**2.02**

Threshold	RR (95% confidence interval) 30°C	32°C	34°C	35°C	35.5°C	36°C	RaRR
Lag2	1.03*** (1.02, 1.05)	1.04*** (1.01, 1.06)	1.01 (0.96, 1.06)	0.95 (0.84, 1.09)	0.98 (0.74, 1.28)	0.47 (0.16, 1.40)	
Age ≥ 65							
Lag0	1.09*** (1.06, 1.13)	1.06** (1.02, 1.11)	1.07 (0.97, 1.17)	1.15 (0.93, 1.42)	1.30 (0.87, 1.93)	1.92 (0.67, 5.52)	
Lag1	1.06*** (1.02, 1.10)	1.07** (1.02, 1.12)	1.09 (0.99, 1.20)	1.16 (0.92, 1.46)	1.62* (1.08, 2.44)	**4.34** (1.43, 13.2)**	**4.09**
Lag2	1.02 (0.99, 1.06)	1.04 (0.99, 1.08)	1.09 (0.99, 1.20)	1.28* (1.02, 1.60)	**1.58* (1.02, 2.43)**	2.65 (0.75, 9.33)	**1.55**

(b) Heat-related hospital visits

Lag0	1.04*** (1.03,1.04)	1.03*** (1.02,1.03)	1.02*** (1.01,1.02)	1.00 (0.98,1.01)	0.97 (0.94,1.01)	**1.27** (1.10,1.46)**	**1.22**
Lag1	1.02*** (1.02,1.02)	1.02*** (1.02,1.02)	1.02*** (1.01,1.02)	1.00 (0.99,1.01)	1.00 (0.97,1.03)	1.00 (0.91,1.09)	
Lag2	1.03*** (1.03,1.03)	1.03*** (1.02,1.03)	1.02*** (1.01,1.02)	1.00 (0.99,1.02)	1.00 (0.97,1.03)	1.03 (0.95,1.13)	
Female							

Threshold	RR (95% confidence interval) 30°C	32°C	34°C	35°C	35.5°C	36°C	RaRR
Lag0	1.03*** (1.03,1.04)	1.02*** (1.02,1.03)	1.01*** (1.01,1.02)	0.99 (0.97,1.01)	0.95* (0.90,1.00)	1.21 (0.99,1.47)	
Lag1	1.02*** (1.02,1.02)	1.02*** (1.02,1.02)	1.02*** (1.01,1.02)	1.00 (0.98,1.02)	0.99 (0.96,1.03)	0.98 (0.88,1.10)	
Lag2	1.03*** (1.03,1.03)	1.03*** (1.02,1.03)	1.02*** (1.01,1.02)	0.99 (0.98,1.01)	0.99 (0.95,1.03)	1.03 (0.92,1.16)	
Male							
Lag0	1.04*** (1.03,1.04)	1.03*** (1.02,1.03)	1.02*** (1.01,1.03)	1.01 (0.98,1.03)	1.00 (0.95,1.06)	**1.36** (1.10,1.68)**	**1.31**
Lag1	1.02*** (1.02,1.03)	1.02*** (1.02,1.03)	1.02*** (1.01,1.02)	1.00 (0.98,1.02)	1.01 (0.97,1.06)	1.01 (0.88,1.16)	
Lag2	1.03*** (1.02,1.03)	1.03*** (1.02,1.03)	1.02*** (1.02,1.03)	1.01 (0.99,1.04)	1.02 (0.98,1.06)	1.02 (0.89,1.17)	
Age 0-14							
Lag0	1.04*** (1.04,1.05)	1.04*** (1.02,1.05)	1.03* (1.00,1.06)	1.05 (0.96,1.14)	1.12 (0.94,1.35)	**2.11* (1.13,3.95)**	**2.03**
Lag1	1.02*** (1.01,1.03)	1.02* (1.00,1.03)	1.00 (0.97,1.03)	0.97 (0.90,1.05)	0.93 (0.79,1.10)	0.93 (0.55,1.57)	
Lag2	1.03***	1.03***	1.04**	1.06	1.06	0.98	

	RR (95% confidence interval)						RaRR
Threshold	30℃	32℃	34℃	35℃	35.5℃	36℃	
	(1.02,1.04)	(1.02,1.05)	(1.01,1.07)	(0.98,1.14)	(0.91,1.24)	(0.58,1.65)	
<u>Age 15-64</u>							
Lag0	1.04***<br>(1.03,1.04)	1.03***<br>(1.02,1.03)	1.02***<br>(1.01,1.02)	0.99<br>(0.98,1.01)	0.96<br>(0.93,1.00)	**1.22***<br>**(1.05,1.43)**	**1.17**
Lag1	1.02***<br>(1.02,1.02)	1.02***<br>(1.02,1.02)	1.02***<br>(1.01,1.02)	1.00<br>(0.99,1.02)	1.00<br>(0.97,1.03)	0.98<br>(0.89,1.07)	
Lag2	1.03***<br>(1.03,1.03)	1.03***<br>(1.02,1.03)	1.02***<br>(1.01,1.02)	1.00<br>(0.99,1.02)	1.00<br>(0.97,1.03)	1.05<br>(0.96,1.14)	
<u>Age ≥ 65</u>							
Lag0	1.03***<br>(1.02,1.04)	1.02***<br>(1.01,1.03)	1.01<br>(0.99,1.04)	0.98<br>(0.92,1.04)	0.98<br>(0.85,1.12)	1.47<br>(0.88,2.45)	
Lag1	1.02***<br>(1.01,1.02)	1.02***<br>(1.01,1.03)	1.01<br>(0.99,1.04)	1.01<br>(0.96,1.07)	1.05<br>(0.94,1.18)	1.32<br>(0.93,1.86)	
Lag2	1.02***<br>(1.01,1.02)	1.01*<br>(1.00,1.02)	1.00<br>(0.98,1.02)	0.98<br>(0.93,1.04)	0.98<br>(0.88,1.09)	0.87<br>(0.60,1.26)	

$^{*}p < 0.05^{**}\ p < 0.01^{***}\ p < 0.001$
