# Supplementary figures and images for: A pilot heat-health warning system co-designed for a subtropical city
Source: PLoS One. 2023 Nov 10;18(11):e0294281. doi: 10.1371/journal.pone.0294281 (PMC10637700; doi:10.1371/journal.pone.0294281)

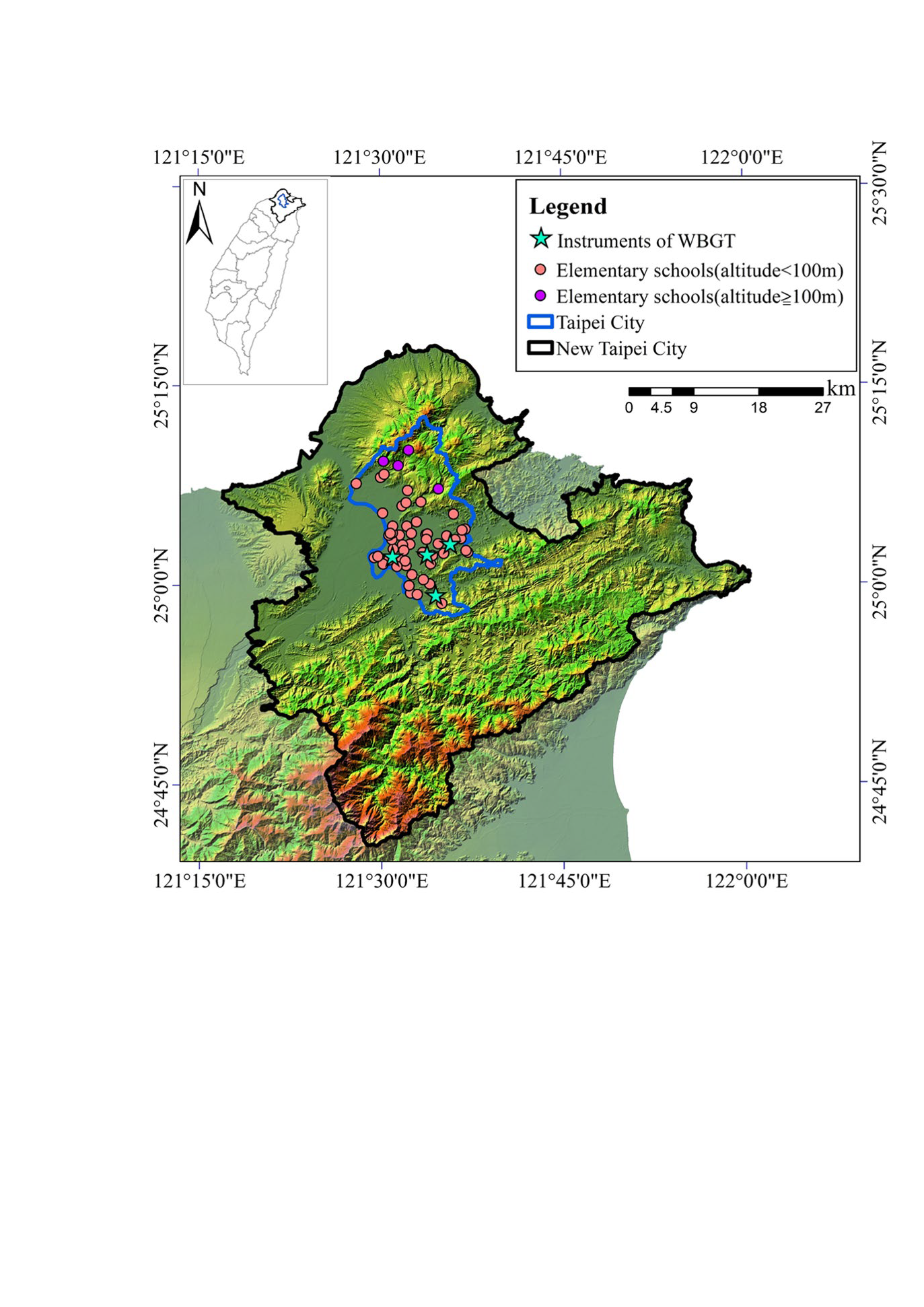

Supplement: S1 Fig — The blue and black lines show the border of Taipei City and the entire Taipei metropolitan area, respectively; the magenta and purple dots are 53 and 4 stations in elementary schools at altitude < and ≥ 100 m, respectively; the star signs indicate the four HOBO monitoring stations of the TPE-pHHWS. Grid numerical terrain model data (20-meter resolution) were obtained from open data [https://data.gov.tw/dataset/35430] under a CC BY license, with permission from Department of Land Administration, Ministry of the Interior, Taiwan, original copyright 2016. Township and urban boundaries were obtained from open data [https://data.gov.tw/dataset/7441] under a CC BY license, with permission from National Land Surveying and Mapping Center, Ministry of the Interior, Taiwan, original copyright 2015. (TIF) [file pone.0294281.s002.tif]

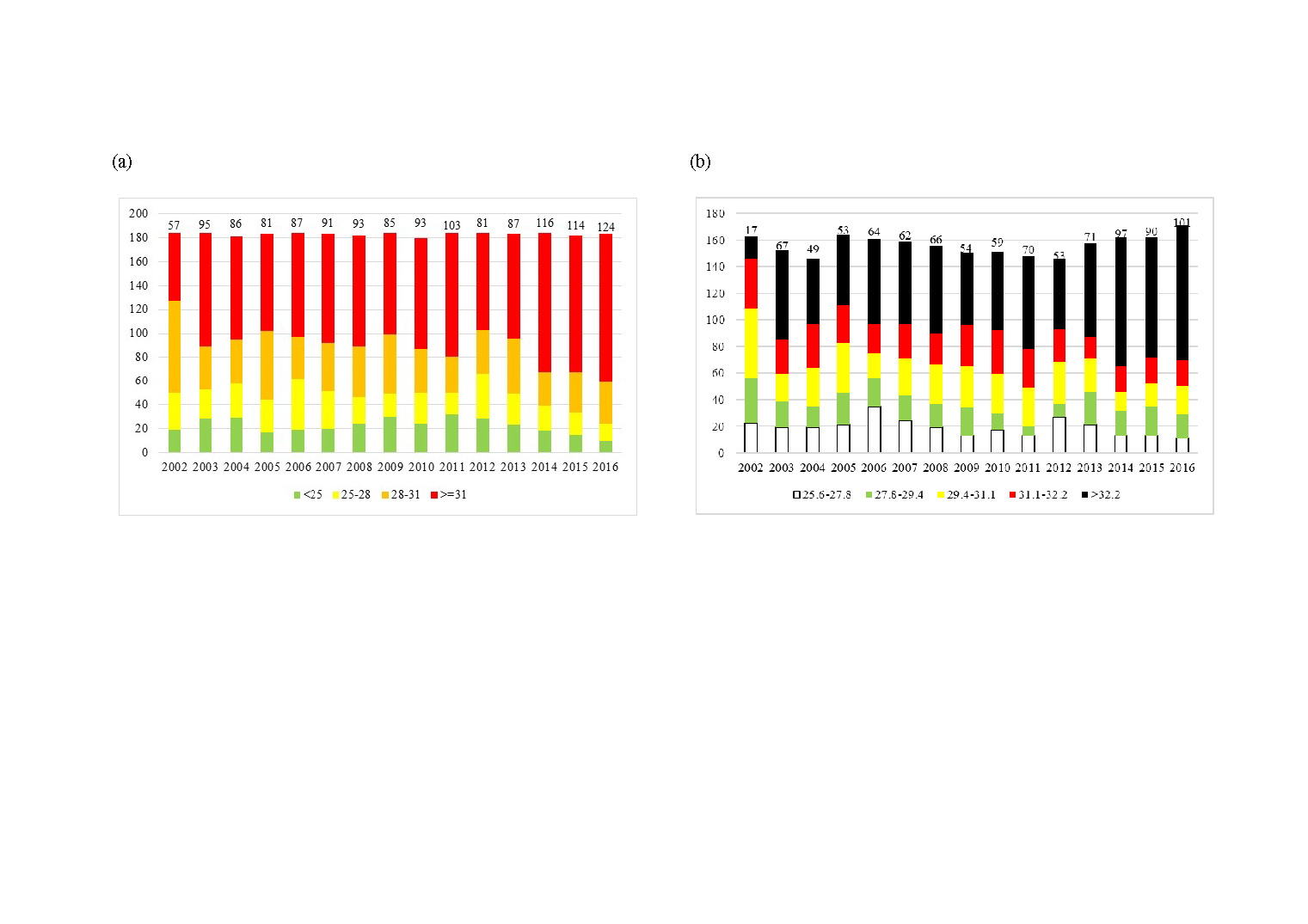

Supplement: S2 Fig — Number of days above thresholds for (a) Japan WBGT categories for the general public and (b) US WBGT categories for the workers. Number of days in the highest WBGT category are listed on the top of the respective column. (TIF) [file pone.0294281.s003.tif]

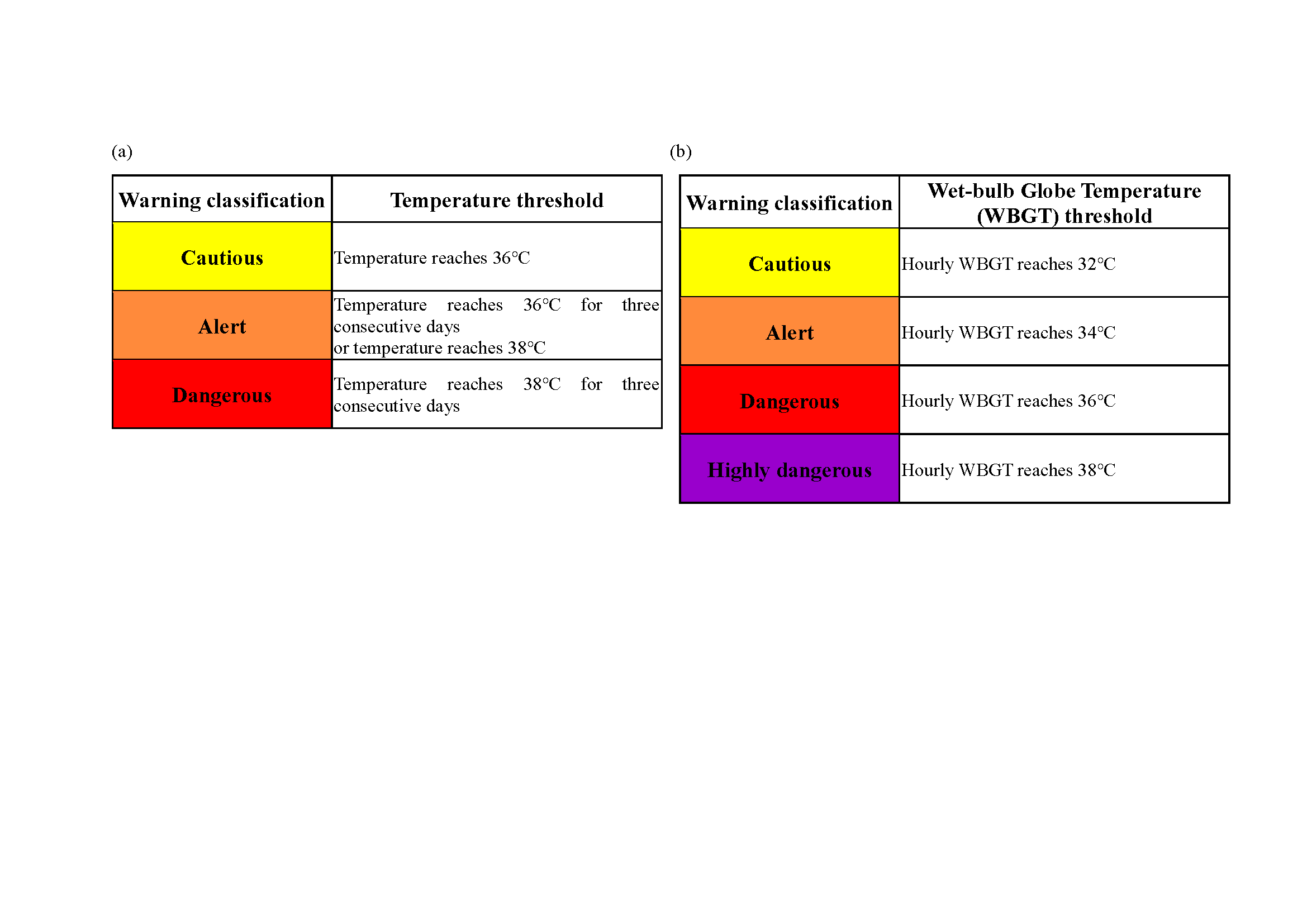

Supplement: S3 Fig — (a) Classification of temperature according to “heat information” announced by Central Weather Bureau (https://www.cwb.gov.tw/V8/E/P/Warning/W29.html (English)) and (b) the classification of Wet-bulb Globe Temperature (WBGT) translated from the data description of https://opendata.cwb.gov.tw/dataset/forecast/M-A0085-001 in Mandarin posted by Central Weather Bureau. Note: The Central Weather Bureau has been upgraded to the Central Weather Administration on September 15, 2023; thus, the webpage may be renamed as www.cwa.gov.tw in the future. (TIF) [file pone.0294281.s004.tif]
